# The Impact of Using Alternative Forages on the Nutrient Value within Slurry and Its Implications for Forage Productivity in Agricultural Systems

**DOI:** 10.1371/journal.pone.0097516

**Published:** 2014-05-15

**Authors:** Felicity V. Crotty, Rhun Fychan, Vince J. Theobald, Ruth Sanderson, David R. Chadwick, Christina L. Marley

**Affiliations:** 1 Institute of Biological, Environmental and Rural Sciences, Aberystwyth University, Gogerddan, Aberystwyth, United Kingdom; 2 Environment Centre Wales, School of Environment, Natural Resources and Geography, Bangor University, Bangor, Gwynedd, United Kingdom; Wageningen UR Livestock Research, Netherlands

## Abstract

Alternative forages can be used to provide valuable home-grown feed for ruminant livestock. Utilising these different forages could affect the manure value and the implications of incorporating these forages into farming systems, needs to be better understood. An experiment tested the hypothesis that applying slurries from ruminants, fed ensiled red clover (*Trifolium pratense*), lucerne (*Medicago sativa*) or kale (*Brassica oleracea*) would improve the yield of hybrid ryegrass (*Lolium hybridicum*), compared with applying slurries from ruminants fed ensiled hybrid ryegrass, or applying inorganic N alone. Slurries from sheep offered one of four silages were applied to ryegrass plots (at 35 t ha^−1^) with 100 kg N ha^−1^ inorganic fertiliser; dry matter (DM) yield was compared to plots only receiving ammonium nitrate at rates of 0, 100 and 250 kg N ha^−1^ year^−1^. The DM yield of plots treated with 250 kg N, lucerne or red clover slurry was significantly higher than other treatments (P<0.001). The estimated relative fertiliser N equivalence (FNE) (fertiliser-N needed to produce same yield as slurry N), was greatest for lucerne (114 kg) >red clover (81 kg) >kale (44 kg) >ryegrass (26 kg ha^−1^ yr^−1^). These FNE values represent relative efficiencies of 22% (ryegrass), 52% (kale), 47% (red clover) and 60% for lucerne slurry, with the ryegrass slurry efficiency being lowest (P = 0.005). Soil magnesium levels in plots treated with legume slurry were higher than other treatments (P<0.001). Overall, slurries from ruminants fed alternative ensiled forages increased soil nutrient status, forage productivity and better N efficiency than slurries from ruminants fed ryegrass silage. The efficiency of fertiliser use is one of the major factors influencing the sustainability of farming systems, these findings highlight the cascade in benefits from feeding ruminants alternative forages, and the need to ensure their value is effectively captured to reduce environmental risks.

## Introduction

Managing nutrients on farms is essential to ensure agroecosystem sustainability, often through the use of nutrient budgeting. Balancing the input and output of nutrients within the farm system is critical to ensuring both short-term productivity and long-term sustainability [1]. The efficient use of feed and fertiliser is central to the sustainability of farming systems. There is a strong impetus that considers animal manure as a source of essential plant nutrients and as a means to improve soil quality [2]–[4], rather than considering it a waste product. Globally, since 2007, agriculture commodity prices rose to historically high levels, leading to concerns about global food availability and food security [5]. Maximising the efficiency of use of nutrients within a system is the key to reducing bought-in fertiliser inputs, which are costly in both economic and environmental terms. Integrating fertiliser use with slurry supply has been known for ∼30 years to be a key way of mitigating and minimising the impact of grazing animals [6]. Life cycle assessment (LCA) studies have suggested a more holistic approach to reduce environmental impacts of farming, improving manure storage, reducing inorganic fertilisers and increasing the use of leguminous forage [7] to reduce the carbon footprint.

Up to 95% of the feed nutrients consumed by ruminant livestock may be excreted in faeces and urine [8]. Therefore, managed correctly, farmyard manure and slurry offer great potential as valuable nutrient balancers, building soil fertility and reducing the need for expensive inorganic fertilisers. The value of fertiliser utilised in the UK is estimated at £1,621 million in 2011, with the value of fertiliser consumed doubling since 2006 [9]. Regular applications of organic fertilisers can also improve both soil structure and condition by increasing water holding capacity, drought resistance, structural stability and biological activity [10]. Using farmyard manure and slurry, provides additional environmental benefits, for example greenhouse gas abatement, and increasing the organic matter content of soils. Adjusting for the fertiliser requirement with manure and slurry, will potentially lead to reductions in inorganic fertiliser application rates [11].

Globally, the increasing demand for animal protein is focusing attention on the source of feed, its suitability, quality and the safety of future supply. It has been estimated that about 1000 million tonnes of animal feed is produced worldwide per annum, and 60% of the world total is from 10 countries [12]. The agricultural feed industry continues to rely heavily on imports of protein for livestock, for example in the UK, the total cost of animal feed rose to £4.4 billion in 2011 [9]. Fluctuations in world feed prices and increasing consumer concerns regarding traceability following numerous crisis's, has led to an upsurge in further demand for home-grown sources of high-quality feed.

The feeding of ryegrass silage often requires the addition of concentrate feed to achieve commercially-viable productivity in ruminants. Advances in silage technology have improved the possibility to ensile alternative forages as high protein winter forage for livestock, giving farmers another option which may reduce their reliance on bought-in concentrate feed. A study comparing consumption of grass and legume silages with concentrates on milk production in dairy cows found higher DM intake and milk yield with the legume silage compared to the grass [13]. In an experiment comparing ryegrass silage to alternatives, lambs offered alternative ensiled forages, notably lucerne and red clover, had a higher dry matter (DM) intake and live-weight gain than lambs offered ryegrass silage [14]. Furthermore, the food conversion and nitrogen (N) use efficiency was higher in lambs offered alternative silages compared with those offered ensiled ryegrass, with lambs offered kale silage having the most efficient use of N. These findings demonstrate the potential for using ensiled alternative forages compared with ensiled ryegrass to improve nutrient use efficiency, and thus, the sustainability of ruminant systems.

In order to determine the effects of incorporating different forages into a livestock system, understanding of the total loss of nutrients by the animal is needed to determine the full economic and environmental impact within a farm nutrient budget plan. Results from earlier experiments with ensiled forages indicate that at least 60% of the N in the forage will be excreted by the ruminant animal [15] resulting in a valuable high N source. This has the potential to replace inorganic N within a farm nutrient plan, reducing the reliance on inorganic N inputs (if correctly stored and applied). Consequently, there is a need to establish the benefits and limitations of integrating different forages into ruminant livestock systems in order to balance efficient production with environmental impact.

Whilst much is known about factors influencing N availability to crops following the application of typical manure types [4], [16], little attention has been paid to the efficiency with which crops can utilise the nutrients from slurries and manures derived from livestock fed alternative forages and their impact on soil nutrient status. Sheep were used as an example of a ruminant system, in an experiment conducted to test the hypothesis that applying slurries from ruminants fed ensiled red clover (*Trifolium pratense*), lucerne (*Medicago sativa*) or kale (*Brassica oleracea*) would alter the yield of swards of hybrid ryegrass (*Lolium hybridicum*) compared with applying slurries from ruminants fed ensiled hybrid ryegrass, or just applying inorganic N alone.

## Materials and Methods

### Ethics statement

The Institute had an ethics committee who meet at regular intervals throughout the year as part of an Ethical Review Process, as required by the Home Office (UK). The experiment reported here did not involve any regulated procedures bound by the Animals (Scientific Procedures) Act 1986 (ASPA) (UK) and did not require Home Office approval. No specific permits were required for this study, because the performance of this study was in accordance with guidelines set by the Institute. No specific permits were required for the described field studies, because the field was owned/managed by the Institute. No specific permits were required for these locations/activities, because the location is not privately-owned or protected in any way and the field studies did not involve endangered or protected species. Ethical considerations made during experiments, related to the nutritional welfare of the sheep kept to obtain slurry for this study.

### Experimental site, plot establishment and maintenance

Twenty-eight field plots (12×2.5 m) of hybrid ryegrass (cv. AberExcel) were sown at the rate of 36 kg ha^−1^ in early September in four replicate blocks, in a randomised complete block design. The plots were sited on an area of stony, well-drained loam of the Rheidol series at the Institute of Biological, Environmental and Rural Sciences (IBERS) site, University of Aberystwyth, Wales (52^o^ 26' 55" N, 4^o^ 1' 27" W) (Table 1 for full details of site characteristics). To achieve an optimal soil pH (of 6.0), ground limestone was applied at the rate of 5 t ha^−1^. Compound fertiliser was applied to achieve phosphate and potash indices of 2+ to 3 [4], muriate of potash at the rate of 140 kg K_2_O ha^−1^ and triple super phosphate at a rate of 100 kg P_2_O_5_ ha^−1^. Plots were treated with the insecticide Dursban 4 (chlorpyrifos 480g l^−1^; Dow Agrosciences, Hitchin, Herts.) applied at 1.5 litres ha^−1^ as a preventative measure as there was likely to be an established population of wireworms (Elateridae) and leatherjackets (Tipulidae) present, prior to sowing. Lupus slug pellets (3% methiocarb; Bayer plc, Bury St Edmunds, Suffolk) were applied at 5 kg ha^−1^ to aid establishment of the ryegrass, as due to the temperate climate (mild and wet) slugs are constantly prevalent (Table 1 for meteorological information). Plots were also treated with a herbicide (UPL Grassland Herbicide, dicamba, 25 g L^−1^; MCPA, 200 g L^−1^; mecoprop-P 200 g L^−1^; United Phosphorus, Warrington, UK) at 5 L ha^−1^. Ryegrass plots were cut in December to a height of 6 cm.

**Table 1:** Site characteristics, previous cropping and initial soil analysis (mean ± standard error).

Location characteristics	
UK Ordinance Survey Grid ref	52^o^ 26' 55" N, 4^o^ 1' 27" W
Altitude (a.s.l.)	30 m
Soil series	Rheidol
Soil type	stony, loam
Annual rainfall (10 year average)	1094 (±54) mm/yr
Drainage status	well-drained
Site history	Grass/Barley
**Initial soil analysis**	
pH (H_2_O)	5.75 (±0.036)
Ammonium-N (mg kg^−1^ DM)	10.1 (±1.15)
Nitrate N (mg kg^−1^ DM)	15.1 (±1.99)
Extractable Phosphorus (ppm)	15 (±2.0)
Potassium (ppm)	90 (±5.7)
Calcium (ppm)	1186 (±35.1)
Magnesium (ppm)	157 (±6.0)
**Weather conditions over two harvest years**	
Average temperature (°C; two year average)	10.6 (±0.83)
Maximum temperature (°C; two year average)	14.0 (±0.86)
Minimum temperature (°C; two year average)	7.1 (±0.82)
Solar radiation (MJ/m^2^/day; two year average)	9.7 (±1.21)
Number of days above 5 °C first harvest year	316
Number of days above 5 °C second harvest year	320
Total rainfall (mm; total first harvest year)	843.2
Total rainfall (mm; total second harvest year)	1101.2
Monthly rainfall (mm; two year average)	81.0 (±7.86)

During the following establishment year, the plots were maintained by cutting to a height of 6 cm on 12 March, 13 May, 25 June, 8 August, 24 September and 10 December and the harvested material removed. Artificial N fertiliser was added to all plots, as 34.5% ammonium nitrate, on 5 occasions: 11 March, 28 March and prior to cuts 2, 3 and 4 to provide a total of 200 kg N ha^−1^ annum^−1^. Potassium and phosphate fertiliser were added as previously, to maintain indices of 2+ to 3.

### Animals and slurry collection

Lambs were used as a ruminant model organism for slurry production, due to size, replication and cost considerations. Slurries were collected from 80 Suffolk-cross finishing lambs fed on ensiled red clover (cv. Merviot), lucerne (cv. Vertus), ryegrass (cv. AberExcel) or kale (Kaleage, a hybrid combining cv. Pinfold and cv. Keeper) during an eight-week period. A description of the feeding experiment during which slurries were obtained was provided in Marley et al., [14]. Prior to slurry collection, the lambs were grouped within gender and according to live weight (mean 30.9 kg (±2.29)) for a six week standardisation period and then adapted to their respective silage treatment over a 14 day period, where the first seven days the alternative forage was introduced as a proportion of the diet (i.e. 0.75∶0.25, 0.5∶0.5, 0.25∶0.75 and 1∶0 of treatment and ryegrass silage offered). A further seven day period with *ad libitum* access to their allocated silage as their sole diet, was permitted for full dietary adaptation. After which, the slurry collection period began, with the lambs continuing to be fed the alternative forage *ad libitum* as the sole diet for eight weeks, whilst slurry was obtained from beneath all 20 lambs within each treatment. Lambs were housed as four replicate groups of five lambs for each treatment (n = 20 per forage treatment) and placed in a sheep housing facility that was arbitrarily divided into four blocks with five pens for each of the replicate groups within each treatment. Mesh flooring placed over plastic trays where used in one of the four blocks and the lambs were rotationally moved every 14 days, in their respective replicated blocks, so that faeces and urine were collected from beneath all 20 lambs within each treatment during the 8 week experiment. Slurry obtained from the different lambs within each treatment was bulked and mixed; however each slurry was kept separate between the individual forage treatments. Each pen of lambs was offered forage *ad libitum*, with feeding levels designed to ensure a refusal margin of 10% each day. Fresh water was available to the lambs at all times. Lambs on red clover, lucerne and ryegrass silage were fed first-cut silage during weeks 1–4 and second-cut silage during weeks 5–8.

### Preparation, storage and the application of slurries and inorganic fertilisers

The faeces and urine collected were diluted initially 1∶1 with water (except kale-fed excreta which was sufficiently dilute) and mixed thoroughly using a ‘Hilta Drysite’ diaphragm pump (Morris Site machinery, Wolverhampton, UK) to form slurries. Slurries were collected over an 8 week period from January – March and stored until required for land spreading. Storage was at 4°C in 1 m^3^ plastic vessels, with a narrow opening at the top and a tap at the base. The vessels were loosely sealed to reduce losses of ammonia nitrogen.

Slurry from animals fed on the four different silages were applied (in addition to 100 kg N ha^−1^ inorganic fertiliser N) to field plots of ryegrass (12×2.5 m per plot) and compared with plots receiving ammonium nitrate at the rate of 0, 100 and 250 kg N ha^−1^ year^−1^, in a randomised block design with a total of 7 treatments in 4 replicate randomised blocks. Slurries were applied manually using calibrated watering cans with a spoon attachment to simulate a splash-plate (surface broadcast) application. At application, the slurries were all diluted so that all slurries were of the same dilution ratio, and were applied at a ratio of 1∶2.5 with water to allow the material to be applied evenly to the plot surface. All slurries were kept well mixed and were the same volume across plots at application; slurry was randomly applied within a set time on the same day to avoid any effects of weather conditions or time of day at application.

Slurries were applied at the rate of 35 t ha^−1^ as a split dressing, with half applied on 26 March and the remainder applied on 20 May, the year following plot establishment. All plots treated with slurry also received ammonium nitrate at 100 kg N ha^−1^ year^−1^ applied as a base application at the rate of 25 kg N ha^−1^ on four occasions (on 18 March, and also immediately after first, second and third cut), using a Gandy plot fertiliser (BLEC Landscaping Equipment Ltd., Spalding, Lincolnshire). Control plots, comprised of plots receiving ammonium nitrate at the rate of 0, 100 and 250 kg N ha^−1^ year^−1^ (to be referred to as 0N, 100N and 250N onwards), ammonium nitrate was applied on the same dates on the solely inorganic N plots as it was applied to slurry-treated plots. Water was applied to all control plots at a rate of 35 t ha^−1^ annum^−1^ on the same dates as slurry was applied, to control variability between treatments. Potassium and phosphate fertiliser were applied as a compound of muriate of potash and triple super phosphate at the rate of 154 kg K_2_O ha^−1^ and 100 kg P_2_O_5_ ha^−1^, to all experimental plots, to ensure neither element was limited during the harvest years.

### Soil and slurry analysis

Preliminary soil samples were taken 15 months after sowing the ryegrass, in the first harvest year prior to slurry application, from a W-formation across each replicate block of each set of plots and bulking each replicate block together (n = 4). Extra samples were taken from the experimental site at each depth to calculate bulk density and water content to allow for the calculation of nutrients per ha.

Experimental soil samples were taken at 0–7.5 cm for mineral analysis, and 0–30 cm and 30–60 cm for N analysis (at some sites bedrock was less than 60 cm from the soil surface, thus less than 30–60 cm depth was taken). Soil analysis was carried out on samples obtained immediately prior to the first slurry application, from cores taken in a W-formation as described above. Further soil analysis was determined from samples obtained six months after the first slurry application and 18 months after the first slurry application, from 6 replicate samples (cores 0–7.5 cm) taken per plot, bulked to form one sample per plot for mineral analysis. Soil samples of 0–30 cm and 30–60 cm were taken for soil N analysis and processed immediately, with soil N being determined as nitrate (NO_3_-N) and ammonium-N (NH_4_-N). Soil mineral analysis (0–7.5 cm cores) was determined for calcium (Ca), magnesium (Mg), potassium (K); and phosphorus (P). Soil P was determined as bicarbonate extractable (Olsen) P and 0.01 M CaCl_2_ extractable P (a measure of potentially mobile P) whilst the other minerals were extracted from soil using acetic acid and measured by inductively coupled plasma (ICP). Soil pH was determined as 1∶1 (soil:water) mixture, shaken for 30 min before the pH was measured.

Sub-samples of each slurry type were collected at the time of spreading and analysed for pH, dry matter (DM) content, total N, nitrate-N and ammonium-N. Ammonium-N and nitrate-N were extracted from slurry using a 2 M KCl solution (10 g slurry in 50 ml KCl shaken for 1 h then filtered). Nitrate was determined by reduction of nitrate to nitrite using a cadmium column followed by colorimetric measurement at 520 nm. Ammonium-N was determined colorimetrically at 660 nm. Total N in slurry samples was determined using a Kjeldahl method (Tecator Kjeltec Auto 1030, Tecator, Höganäs, Sweden). The two-step process involved digesting the sample using sulphuric acid and a digestion catalyst which converts the organic N content to the ammonium form. The sample digest was then analysed for ammonium-N by distillation and titration. DM was determined by drying a known amount at 105 °C for at least 24 hours. The pH was determined after mixing 10 g of slurry with 50 ml deionised water. The solution was allowed to settle for 30 min before the pH was measured.

### Sward density, herbage yield, nitrogen offtake and sward composition

Plant population densities were monitored during the spring and autumn of each year. The mean ryegrass tiller count m^-2^ was determined from eight randomly-placed 12×18.75 cm quadrats per replicate block, in the autumn and spring, post slurry application.

During the first year after slurry application (first harvest year), plots were cut on 18 May, 30 June, 19 August, 12 October and 10 December. In the second harvest year, plots were cut on two occasions – 16 May and 6 July to measure any residual carry-over effects. Plots were harvested using a Haldrup 1500 plot harvester (J. Haldrup a/s, Løgstør, Denmark), and cut to a height of 6 cm. Yield was determined by weighing the material cut from an area of 12 m×1.5 m within each plot. Sub-samples of forage, as harvested, were taken to determine dry matter (DM) yield, N offtake and the botanical composition of the sward. All sample material was stored at −20°C prior to subsequent chemical analysis. The DM contents of the herbage was determined by drying to constant weight at 80 °C in a forced-draught oven, and the DM content of the samples taken for chemical analysis after freeze-drying. Total N of the herbage cut was determined using a Leco FP 428 nitrogen analyser (Leco Corporation, St. Joseph, MI, US).

### Statistical analysis

Effects of fertiliser treatment on plot yields, N balance and recovery were assessed by analysis of variance according to the randomised block design. Differences in the composition of slurry applied to the slurry plots were assessed similarly on the relevant subset of the design. Soil mineral composition on two sampling dates and N content at two depths were compared by split plot analysis of variance with fertiliser treatment effects assessed at the whole plot level and effects of sampling date and/or depth and their interaction with fertiliser assessed at the sub-plot level. Where applicable, multiple comparisons within tables of means were made using the Student Newman Keuls test [17] with the experiment-wise type I error rate set at 5%. The total inorganic fertiliser N equivalence (FNE) of each slurry was estimated by a within-block reverse interpolation assuming a quadratic diminishing response in DM yield across the three inorganic N treatments (including 0 N) (N = 4 per treatment). Slurry N efficiency in terms of DM yield was estimated as total inorganic N equivalence less 100 kg (applied as ammonium nitrate) relative to slurry N applied. To understand the difference in N utilisation for each treatment, the apparent N recovery (ANR) was calculated according to the method of Kanneganti et al., [18]. The N offtake relative to 0N or 100N, was calculated; ANR =  ((NTRT-NCON)/NTOT)*100 where NTRT is N offtake, NCON is N offtake from control and NTOT is total N applied, all measured in kg ha^−1^ yr^−1^ and expressed as a percentage of the difference in total N applied. All data were analysed using GenStat (14th Edition, [19]) and are presented as mean and S.E.D (standard error of the difference), unless otherwise stated.

## Results

### Slurry

Lambs fed on kale silage produced a higher amount of excreta than lambs on other silages (P<0.001), the total dry matter (DM) from lambs fed on kale silage was lower than lambs fed the legume silages, and it also had a significantly lower N content (Table 2). Kale slurry had less than a third of the DM content of all the other slurries applied (Table 2). Lambs fed lucerne and red clover produced an intermediate amount of slurry compared to kale and ryegrass, however these two alternatives had the highest dry matter and N content (both P<0.001) compared to the other slurries. Hybrid ryegrass fed lambs produced the least amount of slurry per day, although ryegrass had lower dry matter and N content. There also were significant differences in composition between the slurries applied (P<0.001) for pH, nitrate and ammonium and total N contents (Table 2). In terms of pH all slurries were significantly different from each other (P<0.05), with ryegrass having the lowest pH and lucerne the highest (Table 2). Lucerne and red clover slurry both had high total-N content, whilst kale had the lowest total-N followed by ryegrass which was intermediate (Table 2). Nitrate N concentration was higher in kale slurry (1.07 mg kg^−1^) than in the remaining slurries (P<0.05) which showed levels <1 mg kg^−1^. The ammonium-N content of the ryegrass and kale slurries were similar and significantly lower than the red clover slurry which in turn was lower than the lucerne slurry. Lucerne slurry showed the highest percentage concentration of ammonium-N compared to all other slurries (P<0.05). Overall, kale slurry was the most different to the legume slurries, with the hybrid ryegrass slurry as an intermediate. All environmental variables were considered to be the same for each treatment, as the replicated plots were all located within the same 100 m^2^ area (Table 1).

**Table 2:** Mean composition of slurries (fresh weight) as applied to plots of hybrid ryegrass.

Forage Fed	Undiluted slurry (per lamb per day) (kg)	N content (%) undiluted slurry	Dry Matter (g kg^−1^)	pH	NO_3_-N (mg/kg)	NH_4_-N (mg/kg)	NH_4_-N (% Total N)	Total N (g/kg)
H. Ryegrass	0.92^a^	0.517^b^	68.1^b^	7.2^a^	0.14^a^	300^a^	9.1^a^	3.35^b^
Kale	2.73^c^	0.404^a^	20.9^a^	8.2^c^	1.07^b^	290^a^	12.1^a^	2.43^a^
Lucerne	1.69^b^	0.882^d^	72.5^c^	8.4^d^	0.29^a^	1688^c^	31.3^c^	5.46^c^
Red clover	1.34^b^	0.740^c^	72.7^c^	8.0^b^	0.19^a^	989^b^	20.4^b^	4.87^c^
s.e.d	0.185	0.0418	0.673	0.036	0.090	69.95	2.19	0.306
Probability	<0.001	<0.001	<0.001	<0.001	<0.001	<0.001	<0.001	<0.001

Analysis of variance was used to assess differences between composition of slurry for all organic fertiliser treatments. Treatment effects were apportioned using a Student Newman Keuls test (different superscripts following mean indicating significant differences (P<0.05) between treatments), N = 4.

### Soil

Looking at the composition of soils after slurry application there was no evidence of interaction between effects of treatment and sampling date for any of the analytes measured (Table 3). This lack of interaction significance was because the general trend appears to be the same across all treatments; between autumn and spring pH, K, and P contents decreased (P<0.01), and Ca and Mg levels increased (P<0.01), suggesting differences in the release rates of essential nutrients over time. The Ca and P contents were not significantly affected by treatment (P = 0.322 and P = 0.333 respectively), however, there were significant differences over time (Table 3). Using an analysis of variance, near significant differences were also noticeable in the pH level of the soil between treatments (P = 0.054). There was a significant difference over time, with all pH's decreasing; due to this trend across treatments the interaction was not significant. The level of soil K was lower with the 250N treatment than with the other treatments (P<0.05). Mg levels were higher in soils treated with legume slurry than the other treatments and were highest for red clover slurry treated soil (P<0.05).

**Table 3:** Mean mineral composition (g kg^−1^) and pH of soils (0–7.5 cm cores) from plots of hybrid ryegrass in the autumn and following spring after application of inorganic N or slurry from lambs offered different silages.

	Sampling	Treatment							*Mean*	Treatment (T)		Sampling (S)		T.S
		0N	100N	250N	HRG	Kale	Lucerne	Red Clover		s.e.d.	Prob	s.e.d.	Prob	Prob
K	Autumn	248	201	142	241	209	207	206	*208^b^*	15.5	<0.001	5.9	<0.001	0.422
	Spring	208	158	137	191	172	166	189	*174^a^*					
	*Mean*	*228^b^*	*179^b^*	*140^a^*	*216^b^*	*190^b^*	*187^b^*	*198^b^*						
Ca	Autumn	1638	1378	1458	1555	1480	1592	1335	*1491^a^*	83.7	0.322	49.3	<0.001	0.547
	Spring	2028	1959	2148	2093	2101	2088	2081	*2071^b^*					
	*Mean*	*1833*	*1668*	*1803*	*1824*	*1791*	*1840*	*1708*						
Mg	Autumn	89	90	88	95	95	101	105	*94^a^*	1.8	<0.001	1.1	0.003	0.327
	Spring	93	97	92	94	95	103	112	*98^b^*					
	*Mean*	*91^a^*	*93^a^*	*90^a^*	*94^a^*	*95^a^*	*102^b^*	*108^c^*						
P	Autumn	45	38	41	42	38	47	43	*42^b^*	2.4	0.333	1.5	<0.001	0.507
	Spring	23	24	27	25	21	24	25	*24^a^*					
	*Mean*	*34*	*31*	*34*	*34*	*30*	*35*	*34*						
pH	Autumn	6.72	6.61	6.65	6.74	6.74	6.85	6.72	*6.72^b^*	0.054	0.054	0.024	0.003	0.657
	Spring	6.64	6.56	6.64	6.65	6.58	6.72	6.67	*6.64^a^*					
	*Mean*	*6.68^ab^*	*6.59^a^*	*6.64^ab^*	*6.70^ab^*	*6.66^ab^*	*6.79^b^*	*6.70^ab^*						

Analysis of variance was used to assess differences between composition of soils for all treatments (T), sampling time (S) and the interaction between treatment and sampling time (T.S). Effects were apportioned using a Student Newman Keuls test (different superscripts following mean indicating significant differences (P<0.05) between treatments). N = 4.

The ammonium, nitrate or total N content of soil at both the 0–30 cm or 30–60 cm depth was assessed at the beginning and end of the growing season, however no differences were found between treatments (P>0.05) (Table 4). However, there were significant differences found between depths for nitrate and total N (both P<0.001) in the autumn, with lower levels in the 30–60 cm sample than the 0–30 cm sample (Table 4). Significant differences were also found between the two different depths after the growing season had finished (P<0.001) for nitrate, ammonium and total N (Table 4).There were significant changes in the soil mineral N content over the growing season, particularly for nitrate and ammonium (P<0.001), with a reduction in nitrate in the top 0–30 cm of soil over the season and an increase in the 30–60 cm layer. Whilst for ammonium there was an increase in both depths over the season. There were no significant changes found depending on treatment and time of sampling, nor was the interaction between treatment, depth and time of sampling significant (Table 4).

**Table 4:** Mean N content of soil (mg kg DM^−1^) from plots (0–30 cm and 30–60 cm) of hybrid ryegrass in the autumn and following spring after application of inorganic N or slurry from lambs offered different silages.

Sampling	Nitrogen	Depth	Treatment							*Mean*	Treatment (T)		Depth (D)		T.D	Sampling (S)		T.S	D.S	T.D.S
			0N	100N	250N	HRG	Kale	Lucerne	Red Clover		s.e.d.	Prob	s.e.d.	Prob	Prob	s.e.d.	Prob	Prob	Prob	Prob
Autumn	NO_3_-N	0–30 cm	4.9	5.6	6.8	5.9	6.6	6.3	6.1	*6.0^b^*	0.40	0.232	0.18	<0.001	0.275	0.18	<0.001	0.655	<0.001	0.326
		30–60 cm	0.4	0.3	0.6	0.4	0.4	0.4	0.4	*0.4^a^*										
	NH_4_-N	0–30 cm	5.3	4.8	4.8	5.2	5.8	5.0	4.9	*5.1*	0.80	0.720	0.52	0.129	0.669	0.22	<0.001	0.828	0.003	0.274
		30–60 cm	3.1	3.7	4.3	5.4	3.4	4.6	5.5	*4.3*										
	Total N	0–30 cm	10.2	10.4	11.6	11.1	12.3	11.3	11.1	*11.1^b^*	0.87	0.392	0.62	<0.001	0.831	0.30	0.929	0.791	<0.001	0.372
		30–60 cm	3.5	4.0	4.9	5.9	3.8	5.0	5.9	*4.7^a^*										
Spring	NO_3_-N	0–30 cm	3.0	2.9	2.6	2.7	2.7	2.3	2.9	*2.7^b^*	0.28	0.669	0.41	<0.001	0.894					
		30–60 cm	0.6	0.5	1.0	1.1	0.3	1.7	1.0	*0.9^a^*										
	NH_4_-N	0–30 cm	8.0	6.6	7.2	6.9	6.6	7.4	7.9	*7.2^b^*	0.78	0.840	0.44	<0.001	0.743					
		30–60 cm	4.3	4.3	5.4	5.5	5.2	5.8	4.9	*5.0^a^*										
	Total N	0–30 cm	10.9	9.5	9.8	9.6	9.3	9.7	10.8	*10.0^b^*	0.91	0.724	0.76	<0.001	0.864					
		30–60 cm	4.9	4.8	6.3	6.6	5.5	7.5	5.9	*5.9^a^*										

Analysis of variance was used to assess differences between N content of soils for all treatments (T), depth (D), sampling time (S) and the interactions between treatment and depth (T.D), treatment and sampling time (T.S), depth and sampling time (D.S), and treatment, depth and sampling time (T.D.S). Effects were apportioned using a Student Newman Keuls test (different superscripts following mean indicating significant differences (P<0.05) between treatments). N = 4.

### Dry matter yields

**Figure 1:**
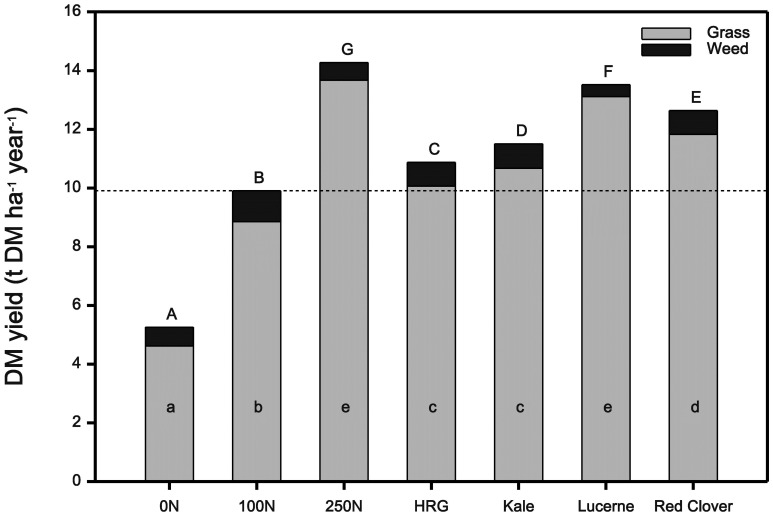
Total annual dry matter yield (t DM ha^−1^ year^−1^) of sown and unsown (weed) species. Plots of hybrid ryegrass treated with slurries from sheep offered four different forage diets (H. ryegrass (HRG), kale, lucerne or red clover) or with inorganic nitrogen at the rate of 0, 100 and 250 kg N ha^−1^ year^−1^, (N = 4). Dotted line indicates yield obtained for the control (100N). There were significant differences between treatments for total yield and sown yield (*P*<0.001). Treatment effects were apportioned using a Student Newman Keuls test looking at total yield (capital letters) and total sown species yield (lowercase letters) indicate significant differences (P<0.05) between treatments. There were no significant differences found between unsown species yield.

Overall DM yields were significantly different between treatments (P<0.001), with all slurry treatments and 250N inorganic fertiliser treatment having significantly greater yields than the 100N and 0N treatments (Figure 1, dotted line representing the “control” 100N yield). Of the different treatments the DM yield increased significantly from ryegrass<kale<red clover<lucerne<250N. Treating plots with slurries from animals fed on different forages or with different levels of inorganic N did not alter the yield of unsown (weed) species (P = 0.121). However the percentage of unsown species (by mass) in total yield was significant (P = 0.001), with 100N and 0N inorganic fertiliser treatments having a significantly greater proportion of unsown species in comparison to lucerne and 250N fertiliser, which had the lowest unsown species proportion. Total ryegrass (sown species) DM yield was significantly different between treatments (P<0.001; Figure 1). All treatments had significantly greater yields than the control (100N); ryegrass and kale slurry had similar yields, which were significantly lower than red clover yields, which was significantly lower than lucerne and 250N sown species yield. There were no significant differences in ryegrass tiller counts between treatments (P = 0.246), or over time (P = 0.569). Nor was there any effect of slurry or fertiliser applications on ryegrass tiller counts taken in two or three years post-sowing.

**Figure 2:**
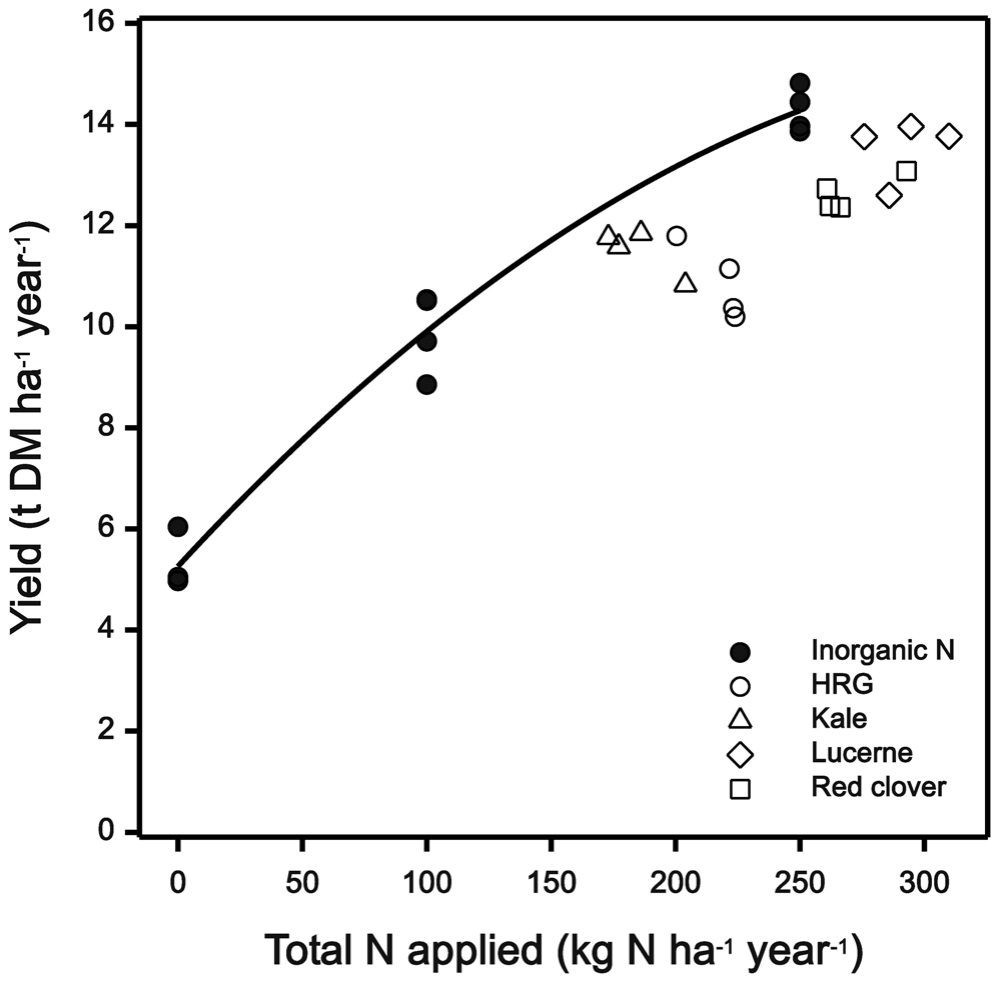
Total annual yield (kg DM ha^−1^) compared to the total N applied (kg N ha^−1^). Plots of hybrid ryegrass treated with slurries from sheep offered four different forage diets (H. ryegrass (HRG), kale, lucerne or red clover) or with inorganic nitrogen at a rate of 0, 100 and 250 kg N ha^−1^ year^−1^, (N = 4). Estimated relative fertiliser N equivalence is indicated by the quadratic regression line.

The DM yield was positively correlated with the amount of N the crops received (Figure 2). Considering that different amounts of N were applied for each treatment (Table 5), this is probably the main factor that contributed to the differences in yield. Overall, when considering the estimated relative inorganic fertiliser N equivalence (FNE) of each slurry and the efficiency of N from the different slurries used to produce DM yield, there were significant differences between treatments. After subtraction of 100 kg inorganic N ha^−1^, the N applied as slurry was equivalent to 114 kg for lucerne, 81 kg for red clover, 44 kg for kale and 26 kg inorganic N ha^−1^ yr^−1^ for ryegrass slurries. Given slurry N application rates of 117, 85, 170 and 191 kg N ha^−1^ in terms of DM yield, these FNE values where significantly greater for all of the alternative forages compared to ryegrass (Table 5). This showed that efficiency of use of ryegrass slurry N for DM yield relative to fertiliser N is lower than that of the other alternative slurries (P = 0.005).

**Table 5:** Mean total N input, offtake and N balance (kg ha^−1^ year^−1^), apparent N recovery (%) and estimated relative fertiliser N equivalence (FNE) for slurry N efficiency, for plots of hybrid ryegrass treated with inorganic N or slurry from lambs offered different silages, (N = 4).

	Total N input(1)	N offtake	N balance(2)	Apparent N Recovery (%)		FNE(5)
				Applied N^(3)^	Slurry N^(4)^	(%)
0N	25	99^a^	−74^a^			
100N	125	165^b^	−40^b^	65^b^		
250N	275	268^f^	7^c^	68^b^		
H. Ryegrass	242^b^	197^c^	45^d^	45^a^	29	23^a^
Kale	210^a^	196^c^	14^c^	52^a^	37	52^b^
Lucerne	317^c^	246^e^	71^d^	50^a^	43	60^b^
Red Clover	296^c^	223^d^	73^d^	46^a^	35	47^b^
s.e.d.	10.9#	8.4	12.9	5.5	8.5	7.7
Prob	<0.001#	<0.001	<0.001	0.003	0.462	0.005

a, ^b^Different superscript letters denote significant differences between means (*P*<0.05).

# relates to means for slurry treatments only

(1) Sum of inorganic N, slurry N plus atmospheric N deposition at a rate of 25 kg ha^−1^ year^−1^ [52].

(2) The N balance was calculated by subtracting offtakes, summed over the entire period (five cuts) from total N input [53].

(3, 4) Apparent N recovery (ANR) was calculated for each plot within each replicate block according to the method of Kanneganti et al., [18] as N offtake relative to 0N (3) or 100N (4), expressed as a percentage of the difference in total N applied. ANR = ((NTRT-NCON)/NTOT)*100 where NTRT is N offtake, NCON is N offtake from control and NTOT is total N applied, all measured in kg ha^−1^ yr^−1^.

(5) Estimated by reverse interpolation assuming a quadratic diminishing response in DM yield across the three inorganic N treatments (including 0 N treatment).

In terms of N yield, offtakes harvested from the different treatments were found to be significantly different between all treatments (P<0.001). The majority of individual treatments had significantly different N offtakes apart from kale and ryegrass which were similar (Table 5). All slurry treatments had greater N offtakes than the 0N and 100N treatments. However, the greatest offtake was the 250N treatment. The nitrogen balance was significantly different between treatments, with the greatest deficit being the 0N input, however 100N input, also had a deficit and both were significantly different to the other N balances. The 250N and all slurry treatments, had a positive N balance; with the ryegrass, lucerne and red clover treatments having the greatest surplus. Apparent N recovery represents the amount recovered by the crop from the fertiliser/slurry. Relative to the 0N treatment, 100N and 250N showed total N recoveries of 65% and 68% while the slurries all showed significantly lower (P<0.05) values, but there was no significant difference between the slurry recoveries, ranging from 45% to 52% (Table 5). Apparent recovery of slurry N calculated by reference to the 100N treatment did not differ significantly (P = 0.462) between the slurries but with the lowest value associated with slurry derived from a ryegrass silage diet (28%) and the highest associated with slurry from lucerne silage diet (43%) (Table 5).

## Discussion

The aim of this experiment was to improve our understanding of the plant-animal-soil nitrogen cycle [20] within livestock production systems. Optimising nutrient supply has the greatest potential to balance intensive livestock production, by converting the detrimental increases in N from animal excreta into a benefit, via the utilisation of slurry, simultaneously, reducing chemical costs and decreasing the environmental impact of farming. Our study illustrates how the use of home-grown alternative forages could reduce the input and output of nutrients within farming systems, thus ensuring both short-term productivity and long-term sustainability. A study looking at the economics of storage, transporting and spreading slurry found that despite high energy costs, it was actually a much lower cost per kg of available N compared to inorganic fertiliser [21]. Previous research has tended to focus on comparison between ranges of fertilisers (form of fertiliser) [22], how they are applied (surface application versus shallow injection) [23] or from which species of livestock they originate [24] but few studies have examined the effects of different forage diets within the same livestock species, or the nutrient value of this as a farm resource.

Understanding the nutrient budgets of farming systems at different scales is central for the efficient use of the available nutrients, to effectively improve the long-term sustainability and environmental impact of farming systems [25]. The utilisation of slurry rather than inorganic fertiliser has the potential to impart large economic value, directly by the reduction in expenditure on inorganic fertilisers and exploiting a natural farm resource. For example, an investigation of the profitability and performance of grazing steers on ryegrass with inorganic fertiliser compared to a ryegrass and legume mix, found no difference in performance but an increased cost of US$19 ha^−1^ for the ryegrass with inorganic fertiliser [26]. Whilst within Europe, it is thought the introduction of legume and grass-legume silages (compared to grass silage) has the potential economic gain of €137 ha^−1^, corresponding to a gain of as much as €1300 million to the European livestock farming sector [27]. Indirectly the use of slurry will also provide a number of ecosystem services, through the changes in soil structure, the direct addition of organic matter and the favouring of different soil food webs [28].

This study used sheep as an example of a ruminant organism for slurry production, due to their size, ease of replication and total cost considerations for the overall experiment involving several treatments. Ruminant research is known to focus on sheep, particularly when using specialised feed to produce slurry e.g. [29]–[30]. However, it is recognised that it is difficult to draw full comparisons between cattle and sheep, given species differences in grazing habits, digestive efficiencies, and intakes [31], however research focusing on the slurry component has found less differences than may normally be expected [32]. One of the main differences between slurries used in the current experiment, before application was the dry matter content and the amount of manure produced. These differences have a long-term management impact; the amount of dilution needed before application, as well as the potential storage issues if these slurries were used in normal farming practice. Although in practice, farmers do not dilute slurries to produce spreadable material, water is added through the washing of housing units and drainage. In the European nitrates directive, 58% of England has been classified as a nitrate vulnerable zone [33], leading to protection measures and stricter control of fertiliser application. The differences in N content in the slurries, could potentially lead to different measures being needed. It should be noted that the ryegrass silage offered to produce the slurries in this experiment had a crude protein content that was 1.6% below the average ryegrass silage produced in the UK in the same season due to weather conditions delaying the silage harvest (see [14]). Therefore, the proportion of ammonium-N to total-N in the ryegrass slurry treatment may have been correspondingly lower than a typical ryegrass slurry treatment.

The chemical composition of the slurries in this study before application, were significantly different (pH, NO_3_-N, NH_4_-N and Total N), however this didn't lead to significantly different N levels within the soil, suggesting the differences were ameliorated by the uptake of the growing crop or lost to the environment. Slurry with a lower pH has a reduced risk of ammonia volatilisation after application, compared to those with a higher pH [34]. In this study, ryegrass slurry was significantly lower than the other treatments, with lucerne having a significantly higher pH than the other slurries. Higher DM content within slurry also poses a greater risk of methane emissions during storage [35], and ammonia volatilisation after application as the slurry does not infiltrate the soil as quickly. Kale slurry had the lowest DM content, thereby posing the least risk compared to the other treatments in this respect. A study investigating the impacts of different slurries on gaseous emissions after spreading, however found kale slurry to have the largest N_2_O emissions (compared to lucerne and ryegrass) [36]. Plant available N varied between the different slurries, with kale slurry having the greatest NO_3_ levels, which is the form most plants absorb N through the root system. However, the lucerne and red clover slurry had the highest NH_4_ levels, which can be readily converted to nitrate in the soil [37]. As nitrate-N is the most susceptible to leaching, these slurries could potentially cause a problem in nitrate vulnerable zones if not correctly managed.

The compositional differences in the slurries likely led to the different mineral levels (e.g. K and Mg) in the soil after slurry application. However, soil mineral levels varied over time, suggesting that there may have been differences in release rate or mobilisation. Significant differences were found in the Mg level in the soil after slurry application, with the greatest found in soil where red clover slurry had been applied, also leading to potential carry-over effects. There was no significant difference between the N content of the soil between treatments after slurry application, reducing the potential for variation in future crops. The overall mineral N content of the soil showed significant differences in depth and over time, these changes were unlikely to be due to the different slurry applications, as N content is known to change with depth [38].

A key finding in this experiment was that of the DM yield of the ryegrass after slurry application. Treating plots of hybrid ryegrass with lucerne slurry (plus 100 kg N ha^−1^) had similar DM yield (sown species) compared with plots receiving 250 kg N ha^−1^ of inorganic N alone. The 250N and lucerne treatments had the greatest positive N balances; however kale slurry had the greatest apparent N recovery, followed by lucerne. Suggesting lucerne slurry could be comparable for ryegrass growth, without loss of yield, to inorganic fertiliser treatments. This is likely to be due to the N in these slurries being more efficiently used. The utilisation of slurry as fertiliser is a common practice but, it should be noted that it is not usually slurry that has been produced from animals fed only on a single forage diet, which was the approach taken here for experimental purposes. Our results show the effect a change of diet can have on slurry and the cascade in effects this could have on production. The estimated relative fertiliser N equivalence (FNE), was greatest for lucerne; although all three alternative forages were greater than ryegrass. The estimated FNE values represented efficiencies of 47–60% for the alternative forages compared to only 22% for ryegrass. It should be noted that the FNE of the slurry is only an estimate based on an assumed diminishing N response curve produced from the three inorganic fertiliser treatments (replicated four times at the same experimental site). Although this does not provide an absolute FNE value for these slurry treatments, it does provide a valid relative value when comparing treatments within the context of this experiment.

Not all N applied to crops is taken up by the plant, some is lost to the environment as ammonia volatilisation or denitrification. However, some N will remain in the soil, in crop residues (roots and non-cut grass) and assimilated into soil microbial biomass. Sampling the N composition of the soils in the spring after slurry application, shows there is N remaining within our soils, with significant differences in the interaction between depth and sampling time. All of the deeper soil samples (30–60 cm) taken in the spring having greater amounts of N (Total N, NO_3_-N, NH_4_-N) generally across treatments, then they did the previous autumn, suggesting the transfer of N further down the soil column.

Applying slurries from ruminants fed on different forages also did not significantly alter the DM yield of unsown (weed) species present in ryegrass swards. In fact, the 100N and 0N inorganic fertiliser treatments had a significantly greater proportion of unsown (weed) species in comparison to the other treatments; it is likely that this is because the ryegrass in these low N treatments could not out-compete the weed species to the same extent, it could in the forage slurry and 250N treatments.

Previous studies have focused on the amount of N excreted following consumption of different forage compositions [39]. Investigations of N uptake and yield of corn amended with slurry of different forage-fed cattle, has also shown variation between different forage slurries [40]. These studies concentrated on cattle and forages commonly fed in the USA like soybean and corn, and found differential effects on soil N mineralisation and plant N uptake after application to soil [41]. Dairy diets are often formulated so that crude protein (CP) levels remain similar, independent of feed; these calculations are based on a total CP value (N×6.25). This approach does not account for differences in the N use efficiency (NUE) of the total CP, and there are differences in NUE occurring among these alternative silages [14]. The higher intakes recorded for ruminants offered legume silages relative to grass silage was attributed to legume silages having a higher passage rate due to higher rumen outflow rate [42]. Using proximate analyses for in vitro digestibility values to predict the nutritive value of legume forages may not be accurate, e.g. [43], who showed the degradability of the CP in vivo was the only reliable method to determine N utilisation efficiencies between forages with similar CP values.

Feeding these alternative forage diets resulted in a higher NUE, whilst being produced from legumes which were grown with minimal N fertiliser addition. This subsequently leads to agricultural benefits which are two-fold, with slurries replacing inorganic N for crops that need it, whilst being produced without any inputs. The value of fertiliser utilised in the UK is estimated at £1,621 million in 2011 [9], if by modifying feeding regime slightly the utilisability of slurry can increase, this would reduce costs to the farming industry, making the farming system more sustainable. Research has shown that there are various factors that can influence the efficient transfer of nitrogen from organic manures to plants. Factors include the total N, readily available N, dry matter content and C:N ratio of the manure [44]; the amount applied, timing of application, application method, rainfall and soil type in the field [4], [45]. In grassland soils, organic manures compared to inorganic fertiliser, are known to increase the organic C, the total N, the activity of decomposers, and the supply of nutrients via the soil food web [46]. Manure slurry has also been found to promote a higher bacterial activity and provide greater mineralisable N compared to inorganic fertiliser [47]. Thus as well as being comparable in yield to inorganic fertiliser, using manure slurry has greater value through the provision of more ecosystem services.

The nutrient composition of manures from different livestock and guidelines on their expected values are available [4]. However, it is recommended that farmers analyse their own manure nutrient compositions, as depending on the forages fed to these animals, they may vary. Our results highlight that slurry from sheep can differ significantly in nutrient value depending on food source and this should be considered as part of routine farm management when slurries are used as fertiliser. However, through Defra's “Farm Practice Survey's” it was found that only 23% of UK farmers tested the composition of their slurry [48]. Future work should focus on encouraging the use of different management systems by farmers (e.g. MANNER and MANNER-NPK [15]) to effectively fertilise crops through slurry spreading.

Modelling the profitability of whole farming systems found variation in fertiliser prices to have a relatively small effect on net margins, largely because this cost comprised a small proportion of total costs [49]. However, it is still a significant amount to be considered, particularly when margins are already low. The agricultural industry continues to rely heavily on imports of protein for livestock production, however the effect of feeding concentrates on the nutrient value of livestock slurry was not the focus of this experiment. This study has highlighted the importance of understanding the nutrient content of manures, and how a change in food source can impact yields of future crops. Farmers need to consider how these differences in slurry could affect plant growth and not base application rates on fixed values per ha, as there will be different N loading rates, and therefore different yield responses. We still need to understand the effect variable nutrients of forage provided to all livestock have on slurry composition and spreading guidelines. The efficiency of fertiliser use is the key to the sustainability of farming systems.

The results of this study has shown that slurry derived from ensiled alternative forages is comparable to inorganic fertiliser, when considering DM yield of a future forage crop (hybrid ryegrass). The use of high-protein alternative forages can reduce the need for expensive amendments, building soil fertility, and improving nutrient efficiency in ruminant livestock systems. Thus, optimising nutrient requirements and maximising nutrient capture and retention within the farming system; resulting in a more beneficial and sustainable scenario for production and the environment, than currently exists. Optimisation of the entire manure management continuum [50]; is key to the development of sustainable livestock production systems [51]. Our data could be used for this purpose – to inform sustainability indices and farm nutrient budgets, including carbon foot-printing on livestock farms aiming to reduce reliance on imported feeds and fertilisers

## Conclusions

Overall, the findings have shown the potential to use slurry from ruminants fed home-grown alternative forages as a valuable fertiliser within livestock systems, and the impact of that at a farm nutrient level on the subsequent use of nutrients within slurries produced – improved N fertiliser equivalence compared to ryegrass only slurries. The utilisation of slurry rather than inorganic fertiliser has the potential to impart large economic value, directly by the reduction in expenditure on inorganic fertilisers and exploiting a natural farm resource. However, the value of these slurries will depend on farmers having suitable storage and spreading facilities, to reduce any potential environmental risks from these higher N-slurries and further highlights a requirement for farmers to implement industry guidelines to regularly measure the N value of their slurry. There is a need to identify and develop strategies that will allow the use of these alternative forage crops to further mitigate the impact of livestock systems on nutrients and carbon cycling at a UK and global scale.
